# Dose-dependent effects of insoluble fibre on glucose metabolism: a stratified post hoc analysis of the Optimal Fibre Trial (OptiFiT)

**DOI:** 10.1007/s00592-021-01772-0

**Published:** 2021-07-12

**Authors:** Stefan Kabisch, Caroline Honsek, Margrit Kemper, Christiana Gerbracht, Ayman M. Arafat, Andreas L. Birkenfeld, Ulrike Dambeck, Martin A. Osterhoff, Martin O. Weickert, Andreas F. H. Pfeiffer

**Affiliations:** 1 Department of Endocrinology, Diabetes and Nutrition, Campus Benjamin Franklin, Charité University Medicine, Hindenburgdamm 30, 12203 Berlin, Germany; 2 Deutsches Zentrum Für Diabetesforschung E.V., Geschäftsstelle Am Helmholtz-Zentrum München, Ingolstädter Landstraße 1, 85764 Neuherberg, Germany; 3 Department of Clinical Nutrition, German Institute of Human Nutrition Potsdam-Rehbrücke, Arthur-Scheunert-Allee 114-116, 14558 Nuthetal, Germany; 4 Human Study Center, German Institute of Human Nutrition Potsdam-Rehbrücke, Arthur-Scheunert-Allee 114-116, 14558 Nuthetal, Germany; 5 Department of Internal Medicine IV, Division of Diabetology, Endocrinology and Nephrology, Eberhard-Karls University Tübingen, Otfried-Müller-Str. 10, 72076 Tübingen, Germany; 6 Institute for Diabetes Research and Metabolic Diseases of the Helmholtz Center Munich at the, University of Tübingen, Otfried-Müller-Str. 10, 72076 Tübingen, Germany; 7 Warwickshire Institute for the Study of Diabetes, Endocrinology and Metabolism, The ARDEN NET Centre, ENETS CoE, University Hospitals Coventry and Warwickshire NHS Trust, Coventry, CV2 2DX UK; 8 Centre of Applied Biological & Exercise Sciences (ABES), Faculty of Health & Life Sciences, Coventry University, Coventry, CV1 5FB UK; 9 Translational & Experimental Medicine, Division of Biomedical Sciences, Warwick Medical School, University of Warwick, Coventry, CV4 7AL UK

**Keywords:** Diabetes mellitus type 2, Prediabetes, Diabetes prevention, Impaired fasting glucose, Stratification, Impaired glucose tolerance, Insoluble dietary fibre, Insulin sensitivity

## Abstract

**Aims:**

As the first long-term RCT on insoluble cereal fibre, the optimal fibre trial demonstrated glycometabolic benefits, confirming cohort studies. The combined study intervention of lifestyle recommendations and supplementation with insoluble oat hulls fibre allows to clarify, which amount of fibre is required for a beneficial effect.

**Methods:**

One hundred and eighty participants with impaired glucose tolerance underwent the one-year PREDIAS lifestyle programme and received a blinded, randomized fibre or placebo supplement for two years. We conducted a regression analyses and cut-off-based tertile comparisons in subjects with full data on dietary compliance (food records and accounted supplement; *n* = 120) after one year, investigating effects on fasting blood parameters, oral glucose tolerance test and anthropometry.

**Results:**

We found a nonlinear inverse relation between fibre intake and change in postprandial 2-h glucose levels, showing a metabolic benefit beyond 14 g and a plateau beyond 25 g of total insoluble fibre per day. 2-h glucose levels improved significantly stronger in both upper tertiles (−0.9 [−1.6;−0.2] mmol/l, *p* = 0.047, and −0.6 [−1.6;0.3] mmol/l, *p* = 0.010) compared to the lowest tertile (0.1 [−1.2;1.1] mmol/l), also when adjusted for changes in bodyweight. Subjects with the highest fibre intake showed superior effects on fasting and postprandial insulin resistance, hepatic insulin clearance, leucocyte count and fatty liver index.

**Conclusions:**

Extending the knowledge on the benefits of insoluble oat hulls fibre, our post hoc analysis demonstrates a dose effect for glycaemia and associated metabolic markers. Further research is needed in order to replicate our findings in larger trials.

## Introduction

Type 2 diabetes mellitus (T2DM) as one of the major non-communicable pandemics of the twenty-first century represents an enormous burden for patients and health systems, being a crucial contributor to morbidity, invalidity and premature death. T2DM onset and progression are widely caused and thereby open to treatment on the basis of lifestyle factors, including but not being limited to food intake and physical activity. Several large prevention trials have demonstrated an enormous potential in reducing diabetes incidence by about 40–60% [1–4].

Obesity—as one common component of T2DM risk factors—is addressed by changes in energy balance. However, there are also specific nutritional factors, which act independently of overweight: saturated fats, alcohol and insoluble dietary fibre [5]. Despite the strong successes of the huge diabetes prevention trials, which used a combination of lifestyle improvements, it is still unclear, to which extent each single factor contributes to both the problem and the solution of rising case numbers for T2DM.

Insoluble dietary fibre—which is predominantly found in cereals, but also legumes—has been identified as protective nutritional element in cohort studies. Soluble fibres—including beta-glucans from cereals and several types of non-digestible carbohydrate polymers from fruits and vegetables—do not appear to lower T2DM risk in most epidemiological analyses, [6, 7] despite a large number of small intervention trials showing short-term antiglycemic effects for viscous (i.e. soluble) fibres at all [8, 9], as well as specific soluble fibres such as beta-glucans, resistant starch and psyllium in particular [10–12].

Randomized controlled trials, replicating the huge epidemiological evidence for insoluble fibre, are sparse [13, 14]. The optimal fibre trial (OptiFiT), conducted between 2010 and 2014, was the first large, long-term randomized controlled trial (RCT) investigating the effect of insoluble oat hulls fibre in subjects with pronounced risk for T2DM. It demonstrated a small effect on HbA1c (secondary outcome), while primary outcomes were not achieved (diabetes incidence) or reached statistical significance in women only (2-h glucose levels). The study also highlighted that lifestyle changes aiming for higher intake of fibre were mainly unsuccessful [15]. Supplementation (or fortification) might therefore lead to stronger benefits, especially for subjects with certain risk factors for diabetes onset [16, 17].

Still under debate is the question, which dosage might be necessary to achieve the full effect. In a meta-analysis of previous cohort studies, a linear relation of continuously lowered T2DM risk was reported between a daily intake of 0–15 g of insoluble cereal fibre and an overproportional benefit beginning at 15 g per day, which does not show signs of “saturation” [18]. The above-mentioned large prevention trials consensually aimed for a daily fibre intake of 15 g per 1000 kcal. It is unclear, if this dosage is realistic to achieve and sufficient to provide the maximum glycaemic benefit [1–4]. Similarly, based on these limited data from cohort and intervention studies, dietary recommendations enforce a daily fibre intake of at least 30 g, half of which should be insoluble fibre [19, 20].

The OptiFiT cohort provides the opportunity to assess the actual intake of insoluble fibre from both daily diet and supplement and to evaluate the relation with between fibre intake and metabolic benefit in a dose-dependent manner. Previous analyses of the trial have strictly separated fibre and placebo group. In OptiFiT, both groups were advised to increase fibre intake from all sources to 15 g/1000 kcal. As excellent compliance to lifestyle recommendation or poor adherence to fibre supplementation could affect the results, we aim for a focussed per-protocol analysis of OptiFiT. We aim to test the hypothesis that—similar to epidemiological evidence—intake of insoluble cereal in our intervention has a dose-dependent impact on the major metabolic outcome of OptiFiT, which is change in postprandial glucose levels.

## Research design and methods

The present paper is a post hoc analysis of OptiFiT, for which details and previous results have been published elsewhere [15]. For the 24-month study, we recruited 180 subjects with impaired glucose tolerance (IGT; 2-h glucose between 7, 8 and 11, 1 mmol/l), the metabolic subtype of prediabetes bearing high risk for T2DM and long-term complications [21]. Fasting blood samples (e.g. lipid profile, liver enzymes, CRP, uric acid, leucocyte count) and anthropometric assessments including bio-impedance analysis (BIA; Nutriguard-MS) were scheduled every 6 months, while 2-h oral glucose tolerance tests (75 g; capillary measurements every half hour) were done once a year. Capillary blood glucose concentrations were measured immediately by using the glucose oxidase method (Super-GL glucose analyser; Dr. Müller, Freital, Germany), and liver enzymes and other routine laboratory parameters were quantified using a Horiba ABX SAS analyser (Montpellier, France). Serum insulin and C-peptide were measured using an ELISA technique (Mercodia, Uppsala, Sweden).

Our 24-month study entailed a modified version of the one-year lifestyle programme PREDIAS, a structured “Treatment and Education Program for Prevention of type 2 diabetes”. Group-based consultations were provided at regular intervals: a core intervention of 8 weekly lessons in the first 8 weeks and booster sessions (4 bi-monthly lessons throughout the following 10 months) [22]. We defined specific goals for change in diet quality in accordance with the recommendations of the German Society for Nutrition (DGE): fat intake < 30 kcal%, intake of saturated fat < 10 kcal%, intake of total dietary fibre > 15 g / 1000 kcal. Additionally, we aimed for an increase in physical activity (240 min / week). In order to achieve the goals for dietary fibre irrespective of supplementation, we recommended frequent ingestion of whole-grain products, legumes, vegetables, fruits, in particular berries. The low-fat aspect was targeted by limiting our subjects to low-fat dairy and meat products, while supporting soft margarines and healthy vegetable oils to improve fat quality.

Dietary status and compliance was assessed by food records every 6 months, covering four consecutive days, including at least one weekend day, which reflected their typical eating pattern. Nutrient intake—including intake of insoluble and soluble fibre from non-supplement food sources—was determined using the nutrition software PRODI® 5.8 based on Bundeslebensmittelschlüssel 3.0 [23]. Inconsistencies and missing information on specific products were clarified by contacting the patients. This assessment included intake of dietary insoluble and soluble fibre. The subjects received a personal feedback on their individual current dietary achievements during the PREDIAS group meetings.

More detailed procedures for approval, registration and recruitment, the inclusion and exclusion criteria as well as the overall study design have been published previously [15]

Considering the typical rate of drop-outs, declining compliance over time and loss of precision when providing food records, we decided to specifically assess the one-year period of lifestyle programme paralleled by supplementation. One hundred and thirty-six subjects completed this first year of intervention. Out of these, 120 subjects provided full data for dietary intake and supplement use at baseline and throughout the intervention, therefore resembling our per-protocol dataset. Completers and non-completers did not differ by age, body weight, BMI, body circumferences or glucose levels, but women were more likely to drop out within the first 12 months compared to men (29% vs. 13%; X^2^ test; *p* =  < 0.05).

### Dietary supplement

A blinded flavoured drinking powder supplement was provided to all subjects and had to be consumed twice daily for 24 months. It contained either 7.5 g of mostly insoluble fibre (oat hull derived; Vitacel OF 560–30; Rettenmaier & Söhne, Holzmuehle, Germany; 70 wt% cellulose, 25 wt% hemicellulose, 3–5 wt% lignin, 2 wt% guar gum) or 0.8 g of this fibre mixture (placebo; mainly waxy maize starch, guar gum and isomaltulose) per serving. Supplements were indistinguishable with respect to texture, taste, colour and odour. Further details on the supplementation procedure, measurements and laboratory parameters have been given elsewhere [15]. Adherence to supplementation was controlled by weighing the supplement tins, which the participants were asked to return with the remaining content after each dispensation period. This allowed us to gradually assess actual fibre intake from the supplement and to investigate dose-dependent effects in contrast to the previously undertaken binary comparison of verum and placebo irrespective of compliance. Intake of insoluble fibre from the supplement was added on top of the dietary intake to result in the total intake of insoluble fibre, which was used as measure for compliance and variable for stratification.

### Calculations

Fasting (i.e. mainly hepatic) insulin resistance was assessed by the homeostasis model assessment HOMA_IR_ [24] and the ISI_ffa_ [25], which integrate glucose, insulin and free fatty acid levels. Dynamic insulin sensitivity index was covered by the Cederholm index [26]. We also assessed the hepatic insulin clearance (HIC) according to the established formula [27]. The presence of NAFLD was estimated by fatty liver index, liver fat content was approximated by the liver fat formula by Kotronen et al. [28, 29].

### Statistical analyses

We conducted an exploratory regression analysis to identify a plausible relation between actual total intake of insoluble fibre after one year and the absolute one-year change in capillary 2-h glucose levels. We chose this outcome as it was the primary and strongest responding outcome in core publication of OptiFiT [13]. The best fitting regression curve was identified based on visual inspection of the scatter plot and level of significance. We restricted our analysis to the first year of intervention in order to limit the bias due to diabetes-related drop-outs, most of which occurred after 12 months. For our cut-off analysis, we decided against using pre-defined fibre cut-offs from current dietary recommendations, as these are mainly founded on cohort data. Instead, we compared tertiles of actual fibre intake, for which normal distribution was determined by using the Kolmogorov–Smirnov test. Given the frequent absence of normal distribution, we consistently used Kruskal–Wallis and post hoc Mann–Whitney tests for cross-sectional comparisons and Wilcoxon tests for longitudinal comparisons to assure uniform data presentation. All data are shown as means ± standard deviation. The results were considered significantly different if *p* < 0.05. All statistical analyses were performed using SPSS for Windows program version 25.0 (SPSS Inc, Chicago, IL, USA).

## Results

Completers of the first year of intervention with valid dietary data (*n* = 119﻿) did not differ significantly from completers without dietary data (*n* = 18) with respect to age, sex, body weight or glycaemic levels. Men were more likely to be completers (*n* = 137; 38% males) than non-completers (*n* = 43; 19% males), but age, body weight or glycaemia did not differ between subjects reaching the one-year visit and those who did not. (Data not shown.) The entire analysis for this publication was therefore done in completers with valid dietary data (*n* = 119).

We first analysed, if fibre intake or change in fibre intake were significantly associated with change in capillary 2-h glucose levels, the main outcome of OptiFiT. When plotting total intake of insoluble fibre (level after one year of supplementation) against change in capillary 2-h glucose levels, visual inspection of different regression curves identified an inverse relationship as the best fitting curve (change in capillary 2-h glucose (mmol/l) = (22.67 (mmol/l/g)/fibre intake (g)) – 1.62 (mmol/l); *p* = 0.006). An adjusted model using sex, age, 2-h glucose at baseline and body fat content (BIA) as fully included or step-wise excluded covariates was still significant (*p* = 0.011). None of the covariates had a significant impact on the outcome.

**Fig. 1:**
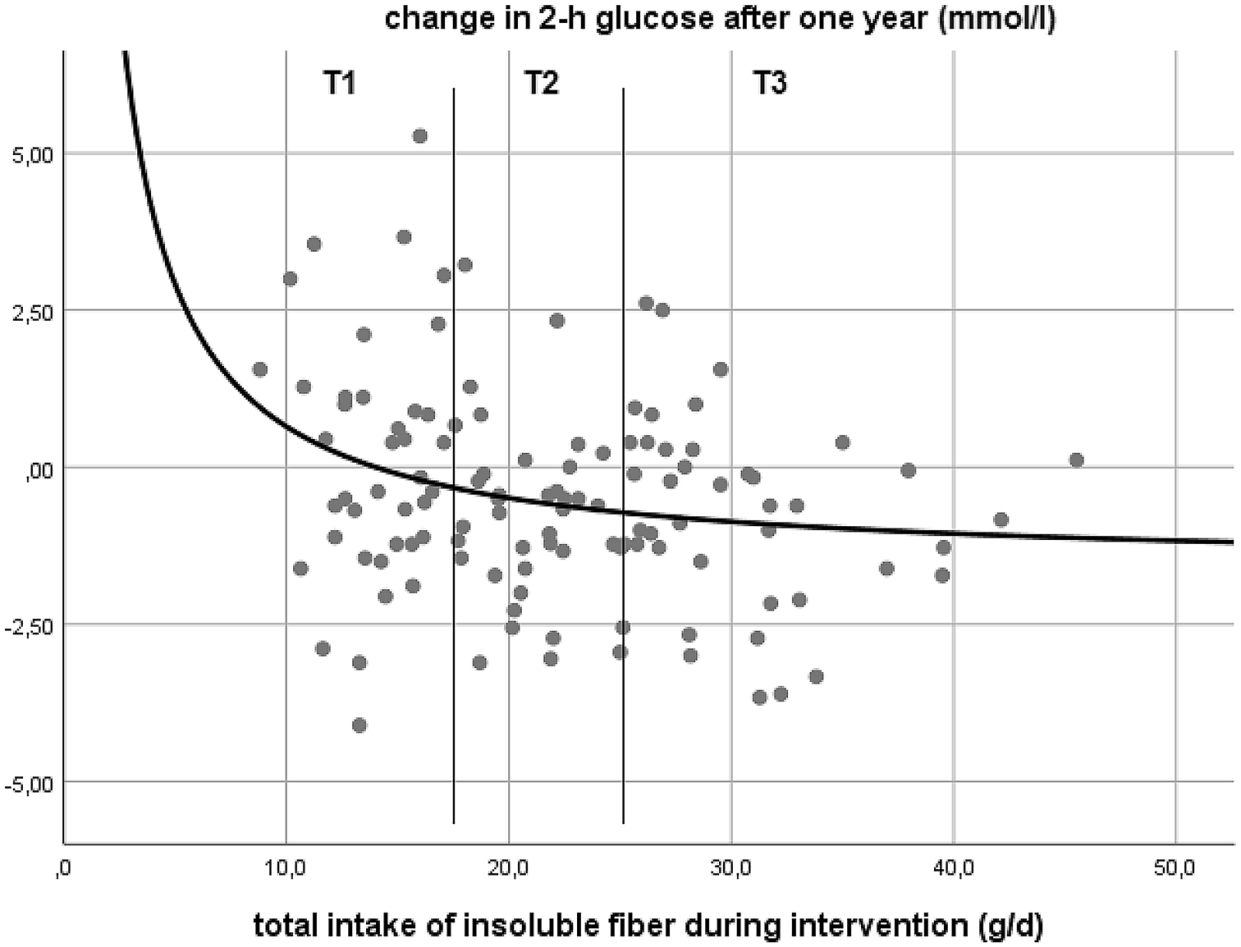
Relation between total insoluble fibre intake during intervention and change in capillary 2-h glucose levels; change in capillary 2-h glucose (mmol/l) = (22.67 (mmol/l/g) / fibre intake (g))—1.62 (mmol/l); *p* = 0.006. T1 and T2: tertile cut-offs for total intake of insoluble fibre

This curve surpasses a neutral metabolic outcome (± 0 mmol/l change in capillary 2-h glucose) towards benefit at 14 g of insoluble fibre and reaches a plateau beyond 25 g, which corresponds to an average reduction in capillary 2-h glucose of about 0.8 mmol/l. (Fig. 1) When plotting total intake of insoluble fibre (level after one year of supplementation) against 2-h glucose levels after one year, an inverse relation of similar shape was the resulting best fitting curve. Choosing instead total fibre intake per 1000 kcal or average intake of insoluble fibre throughout the entire year of intervention as independent variable led to a similar curve. When using *changes* of fibre intake as independent variables, we did not find a significant correlation of any kind. (Data not shown.)

As Fig. 1 shows, a characteristic distribution of all patients within a range of 30 g of fibre intake but different range of metabolic outcome depending on the respective dosage, we additionally decided to define cohort tertiles based on the one-year level of total intake of insoluble fibre (both from daily diet and supplement) compared the respective upper and lower tertiles: tertile 1: 8.8–17.6 g, tertile 2: 17.7–25.2 g, tertile 3: 25.5–45.5 g.

Baseline conditions for these comparisons are presented in Table 1, indicating the tertiles with the highest and lowest intake of insoluble fibre after one year of intervention. There are no significant differences between the respective fibre groups compared to their placebo counterparts with the exception for a significantly lower leukocyte count in the lowest compared to the middle tertile. Within the upper tertile, five subjects belonged to the placebo group and thereby achieved a very high intake of insoluble fibre just on the basis of improved diet. Within the lower tertile, two subjects were allocated to the fibre intervention, but—due to lack in adherence to the supplement—remained at a very low level of fibre intake.

**Table 1:** Characteristics of participants at study entry (split by tertiles for intake of insoluble fibre after one year)

	Tertile 1	Tertile 2	Tertile 3	*p*-value
Age	60 [53;69]	60 [53;67]	61 [58;68]	n.s
Sex (female)	52% (*n* = 21)	65% (*n* = 26)	65% (*n* = 26)	n.s
Weight (kg)	89.0 [76.9;106.2]	92.8 [78.6;106.5]	85.0 [75.0;96.0]	n.s
WHR	0.95 [0.87;1.01]	0.90 [0.86;0.97]	0.94 [0.86;0.99]	n.s
BIA—Body fat (%)	35.1 [28.7;40.7]	39.3 [31.9;42.4]	39.0 [28.5;42.2]	n.s
RR syst. (mmHg)	141 [125;153]	142 [130;153]	138 [130;148]	n.s
Fasting glucose (mmol/l)	5.0 [4.7;5.6]	5.1 [4.8;5.4]	5.0 [4.6;5.3]	n.s
2-h glucose (mmol/l)	8.7 [8.3;9.8]	8.7 [7.8;9.4]	8.4 [8.2;9.6]	n.s
HbA_1c_ (mmol/mol)	37.7 [35.5;39.9]	37.7 [35.5;41.0]	37.7 [34.4;39.9]	n.s
Fasting Insulin (mU/l)	9.42 [7.12;11.93]	7.91 [5.56;11.17]	7.97 [6.00;9.81]	n.s
Fasting C-Peptide (µg/l)	1.55 [1.26;2.07]	1.54 [0.95;2.02]	1.44 [1.17;1.87]	n.s
HOMA-IR	2.4 [1.7;3.2]	2.1 [1.5;3.3]	2.1 [1.6;2.9]	n.s
ISI_ffa_	0.85 [0.62;1.01]	0.94 [0.69;1.19]	0.90 [0.74;1.04]	n.s
Cederholm index	31.9 [26.7;45.3]	39.1 [31.7;43.6]	35.0 [28.2;41.3]	n.s
HIC_c-peptide_ (mU/µg)	4.6 [3.3;5.8]	5.0 [4.4;5.9]	4.2 [3.4;5.0]	n.s
HDL cholesterol (mmol/l)	1.23 [1.01;1.45]	1.22 [1.07;1.40]	1.24 [1.13;1.41]	n.s
LDL cholesterol (mmol/l)	3.56 [3.05;3.90]	3.65 [2.97;3.95]	3.77 [3.15;4.46]	n.s
CRP (mg/l)	2.2 [0.9;5.1]	2.0 [1.3;4.6]	1.9 [0.7;5.0]	n.s
Leukocyte count (Gpt/l)	5.2 [4.3;6.2]	5.7 [5.1;7.0]	5.4 [4.9;7.1]	0.040*
Uric acid (µmol/l)	357 [267;430]	323 [275;389]	325 [300;367]	n.s
Fatty liver index	83 [60;94]	75 [59;93]	74 [32;91]	n.s
Estimated liver fat	5.24 [4.00;7.71]	4.52 [3.13;5.48]	4.75 [2.76;6.51]	n.s

Characteristics of participants at study entry; significant differences between the groups: *p ≤ 0.05, **p ≤ 0.01, ***p ≤ 0.001. (Kruskal–Wallis Tests); Data are median [IQR]. *WHR* waist-to-hip ratio, *HOMA-IR* HOMeostasis assessment of Insulin Resistance, *ISI*_*ffa*_ Insulin Sensitivity Index, using free fatty acids (Belfiore); *HIC* hepatic insulin clearance, *CRP* C-reactive protein

At baseline, dietary patterns with respect to intake of calories and macronutrients from conventional food did not differ between the tertiles. During the intervention, however, as intended, there were strong between tertile differences in the change of intake of dietary fibre from food sources and the supplement, in particular of insoluble oat hulls fibre. Additionally, the middle tertile of fibre intake had a significantly stronger reduction in protein intake (Table 2), which we cannot explain. The strongest impact on fibre intake can be attributed to the supplement, as the placebo group had a marginal rise in insoluble fibre intake from 15 ± 5 to 17 ± 5 g compared to the fibre group (15 ± 5 to 27 ± 6 g).

**Table 2:** Baseline and one-year changes of dietary intake (split by tertiles for intake of insoluble fibre after one year)

	Tertile 1	Tertile 2	Tertile 3	p (T1 vs. T2)	p (T1 vs. T3)
Total energy intake (kJ/day)	8548 [6460;9853]	8222 [7339;10079]	8188 [6996;9820]	n.s	n.s
Carbohydrate intake (g/day)	207 [175;243]	229 [186;274]	221 [174;263]	n.s	n.s
Carbohydrate intake (kJ%)	45 [42;50]	46 [43;49]	47 [41;51]	n.s	n.s
Fat intake (g/day)	81 [57;100]	76 [60;96]	79 [63;98]	n.s	n.s
Fat intake (kJ%)	37 [32;43]	35 [32;40]	38 [32;41]	n.s	n.s
Protein intake (g/day)	76 [64;98]	84 [71;101]	72 [62;92]	n.s	n.s
Protein intake (kJ%)	17 [14;19]	17 [14;20]	15 [14;18]	n.s	n.s
Dietary fibre intake (g/4184 kJ)	11 [9;14]	12 [9;14]	11 [10;14]	n.s	n.s
Total dietary fibre intake (g/day)	21 [18;25]	22 [17;26]	23 [18;30]	n.s	n.s
Insoluble	14 [11;16]	14 [11;18]	15 [13;19]	n.s	n.s
Soluble	7 [6;8]	7 [6;9]	7 [6;9]	n.s	n.s
Steps per day	5817 [4031;7677]	5622 [4477;7057]	6037 [4147;8516]	n.s	n.s
Energy expenditure by steps (kJ)	1400 [975;2304]	1620 [1266;2131]	1614 [1210;2552]	n.s	n.s
*Change during one year*					
Total energy intake (kJ/day)	− 1035 [− 2885;625]***	− 1230 [− 2421;245]**	− 842 [− 1558;433]*	n.s	n.s
Carbohydrate intake (g/day)	− 20 [− 63;31]*	− 10 [− 59;28]	− 7 [− 26;30]	n.s	n.s
Carbohydrate intake (kJ%)	1 [− 4;6]	4 [− 1;8]**	2 [− 2;9]*	n.s	n.s
Fat intake (g/day)	− 8 [− 36;7]**	− 19 [− 27;1]**	− 16 [− 29;12]*	n.s	n.s
Fat intake (kJ%)	− 1 [− 5;3]	− 2 [− 6;3]	− 2 [− 9;4]	n.s	n.s
Protein intake (g/day)	− 3 [− 22;16]	− 17 [− 30; − 2]	− 5 [− 17;6]	0.017*	n.s
Protein intake (kJ%)	2 [− 0;4]***	− 1 [− 3;2] ***	1 [− 2;2]	0.001**	0.0313*
Dietary fibre intake (g/4184 kJ)	2 [− 1;4]	5 [3;8]***	9 [8;14]***	< 0.001***	< 0.001***
Total dietary fibre intake (g/day)	− 1 [− 5;3]	7 [3;10]***	15 [10;20]***	< 0.001***	< 0.001***
Insoluble	0 [− 2;2]	7 [4;10]***	15 [11;18]***	< 0.001***	< 0.001***
Soluble	− 1 [− 2;1]**	− 1 [− 2;2]	1 [− 1;2]*	n.s	< 0.001***
Steps per day	− 630 [2541;889]	703 [− 1372;2088]	135 [− 1598;2813]	n.s	n.s
Energy expenditure by steps (kJ)	− 178 [− 710;192]	134 [335;910]	70 [− 454;464]	n.s	n.s

Baseline status and changes in lifestyle habits during intervention; subjects are split by tertiles for intake of insoluble fibre after one year. Data are median [IQR]. Nutrient intakes were calculated from four-day food records. Physical activity was derived from one-week assessments with pedometers. *Significant changes within the groups (Wilcoxon tests) or differences between the groups (Mann–Whitney Tests): *p ≤ 0.05, **p ≤ 0.01, ***p ≤ 0.001

When comparing interventional metabolic outcomes, both upper tertiles of total insoluble fibre intake at the one-year visit showed a significantly stronger reduction in capillary 2-h glucose levels when compared to the lowest tertile. Inverse regression analysis reveals a significant association between tertile and change in 2-h glucose level (*p* = 0.008). Additionally, the third tertile achieved a significantly stronger improvement in fasting insulin sensitivity (ISI_ffa_), postprandial insulin sensitivity (Belfiore index), HIC, leukocyte count and FLI. (Table 3).

**Table 3:** Outcomes after one year (split by tertiles for intake of insoluble fibre after one year)

	Tertile 1	Tertile 2	Tertile 3	*p *(T1 vs. T2)	*p* (T1 vs. T3)
Allocated to fibre group	2/40 (5%)	23/39 (58%)	35/40 (88%)	< 0.001	< 0.001
Weight (kg)	− 2.0 [− 3.9;0.7]**	− 2.2 [− 5.1; − 0.5]***	− 2.3 [− 5.7;0.1]***	n.s	n.s
WHR	− 0.01 [− 0.04;0.02]	− 0.00 [− 0.04;0.02]	− 0.00 [− 0.03;0.03]	n.s	n.s
BIA – Body fat (%)	− 0.3 [− 2.2;2.2]	− 0.6 [− 2,9;1,4]	− 1.4 [− 6.9;1.8]	n.s	n.s
RR syst. (mmHg)	0 [− 14;13]	− 1 [− 11;5]	− 3 [− 15;7]	n.s	n.s
Fasting glucose (mmol/l)	0.0 [− 0.3;0.3]	− 0.1 [− 0.5;0.2]	− 0.0 [− 0.5;0.3]	n.s	n.s
2-h glucose (mmol/l)	0.1 [− 1.2;1.1]	− 0.9 [− 1.6; − 0.2]***	− 0.6 [− 1.6;0.3]**	0.010*	0.047*
HbA_1c_ (mmol/mol)	0.1 [− 0.2;0.4]	0.2 [− 0.2;0.5]	− 0.1 [− 0.4;0.2]	n.s	n.s
Fasting Insulin (mU/l)	− 0.94 [− 2.76;1.03]	− 1.00 [− 3.13;1.19]*	− 2.24 [− 5.09;0.62]**	n.s	n.s
Fasting C-Peptide (µg/l)	0.08 [− 0.34;0.46]	− 0.04 [− 0.56;0.19]	− 0.09 [− 0.36;0.24]	n.s	n.s
HOMA-IR	− 0.4 [− 0.9;0.3]	− 0.4 [− 0.9;0.3]**	− 0.5 [− 1.3; − 0.1]**	n.s	n.s
ISI_ffa_	0.03 [− 0.12;0.17]	0.05 [− 0.15;0.26]	0.15 [− 0.00;0.42]***	n.s	0.012*
Cederholm index	3.6 [− 5.5;10.3]	7.2 [− 2.9;16.7]**	9.6 [2.2;19.8]***	n.s	0.001**
HIC_c-peptide_ (mU/µg)	1.0 [− 0.5;1.8]	1.4 [− 0.0;2.6] ***	1.7 [− 0.4;2.7]**	n.s	0.041*
HDL cholesterol (mmol/l)	− 0.01 [− 0.10;0.10]	− 0.00 [− 0.12;0.09]	− 0.04 [− 0.12;0.04]	n.s	n.s
LDL cholesterol (mmol/l)	− 0.17 [− 0.38;0.30]	− 0.07 [− 0.34;0.34]	− 0.37 [− 0.64;0.15]*	n.s	n.s
CRP (mg/l)	− 0.6 [− 1.9;0.5]*	− 0.5 [− 1.3;0.6]	− 0.2 [− 1.8;0.3]*	n.s	n.s
Leukocyte count (Gpt/l)	0.3 [− 0.2;0.8]*	− 0.4 [− 0.8;0.3]	− 0.5 [− 1.3;0.3]**	n.s	0.001**
Uric acid (µmol/l)	− 13 [− 43;26]	− 7 [− 49;27]	− 23 [− 55;27]	n.s	n.s
Fatty liver index	− 1 [− 6;6]	− 4 [− 7;1]*	− 5 [− 22;1]***	n.s	0.021*
Estimated liver fat	− 0.91 [− 2.99;0.67]	− 0.89 [− 2.34;0.38]**	− 1.63 [− 3.73;0.02]***	n.s	n.s

Outcomes during intervention; Data are median [IQR]. *Significant changes within the groups (Wilcoxon tests) or differences between the groups (Mann–Whitney Tests): *p ≤ 0.05, **p ≤ 0.01, ***p ≤ 0.001. WHR: waist-to-hip ratio; HOMA-IR: HOMeostasis assessment of Insulin Resistance, ISI_ffa_: Insulin Sensitivity Index, using free fatty acids (Belfiore); HIC: hepatic insulin clearance; CRP: C-reactive protein

## Discussion

Our compliance-based analysis supports previous cohort studies, which reported a beneficial association of higher intake of insoluble oat hulls fibre on diabetes risk [6, 7, 11]. In particular, we can confirm our recent publication on the same data set, which compared IGT subjects based on their allocation to a blinded oat hulls fibre supplement [8]. In the present methodological approach, fibre intake irrespective of group allocation was focussed on, but showed similar results. As in our previous papers, we demonstrate that the improvement in glycaemia is accompanied by an amelioration of fasting and postprandial insulin resistance, also reflected by increased hepatic insulin clearance, and a reduced level of liver fat markers. In addition to that our study highlights that a daily dosage of at least 14 g of total insoluble fibre is necessary in order to achieve a metabolic benefit, confirming current recommendations.

Our publication indicates that achieving a certain level of insoluble fibre intake is more important than just increasing the amount even on top of sufficient baseline levels. Somehow, the beneficial effect seems to undergo a saturation plateau, which contradicts epidemiological data on a steady decline of diabetes with higher levels of fibre intake [6, 11].

We demonstrate that the level of fibre intake after one year of lifestyle intervention and fibre supplementation is mainly independent of the baseline dietary state of the subjects. A strong health belief can affect the adherence to a certain treatment which is perceived as particularly effective by the patients [30]. In our study, most subjects failed to increase their fibre intake by means of regular diet, which was shown in earlier intervention trials [31]. The majority of our patients surpassed the desired level of insoluble fibres by supplementation only.

Previous diabetes preventions trials such as the Da Qing Study, the Indian Diabetes Prevention Programme, the Diabetes Prevention Programme and the Diabetes Prevention Study aimed for an intake of 15 g of total fibre per 1000 kcal [1–4]. Our data show that this target is valid, even though most of the glycaemic benefit seems to be achieved at a lower dose, and sole dietary advice is an insufficient measure to reach an acceptable level.

In confirmation of our earlier analyses of OptiFiT, we show that the glycaemic improvement is driven by reduced insulin resistance [8, 9, 13]. The exact mechanism leading to better insulin sensitivity is not well-understood. Based on an earlier RCT on *pro*teins and insoluble *fibre* in participants with *Met*abolic Syndrome (“ProFiMet”), the role of the intestinal absorption of branched-chain-amino acids—potent activators of the mTOR pathway—was discussed [32] In OptiFiT, faecal samples were not collected. In ProFiMet, also, the involvement of bile acids was investigated [33]. Up to now, we did not assess bile acid levels in OptiFiT. However, we noted that the tertile with the highest fibre intake and best metabolic outcome did also show a stronger improvement in fatty liver index. Even though the ProFiMet study—with healthier subjects—did not report an effect on liver fat, this finding should be followed up in novel trials. The putative role of changes in the gut microbiome cannot be answered by our trial. Our fibre supplement is mainly unfermentable; therefore, we do not expect a mechanistic contribution of short-chain fatty acids. Even apart from that, insoluble fibre could alter the bacterial balance, but without faecal samples, this assumption remains speculative.

Weight loss does not seem to explain the metabolic differences of the three tertiles, as they do not differ in their change of body weight or body composition.

Once again, this analysis, too, demonstrates an anti-inflammatory effect of insoluble oat hulls fibre, which was shown in our recent stratified approach based on obesity [10]. Cohort studies have indicated that high intake of fibre—in particular insoluble fibre and fibre of cereal origin—is associated with lower risk for certain inflammatory disorders [34]. In the context of obesity, metabolic syndrome and prediabetes, insoluble fibre might act by specifically reducing inflammatory processes in visceral adipose tissue.

Increased fibre intake in the context of regular nutrition can be accommodated with changes in the overall dietary pattern and weight loss [22]. We did not see relevant or significant differences between the highest and lowest tertile with respect to changes in macronutrient composition, energy intake or weight loss. Besides nutrition, increased physical activity can lead to metabolic benefits [35]. However, none of the tertiles achieved a significant increase in daily steps or energy expenditure by walking over the first 12 months of intervention.

We like to address some limitations of our work. Selection of completers with full dietary data might contain a selection bias, even though we did not find baseline differences between completers with and without usable food protocols or completers and non-completers. Our cohort of 119 subjects is of moderate size, allowing the conducted comparisons. As a common feature in nutritional RCTs, male subjects are underrepresented.

Our study was conducted with a well-defined insoluble fibre supplement from natural plant origin. However, the metabolic impact may depend on the type of cereal and its processing, leading to differences in particle size, specific composition and food matrix. Replication studies with different supplements and fortified food products are required.

By using 4-day food records and drug accounting of supplement tins, we are able to evaluate dietary compliance on the basis of regular diet and additional fibre intake. There are no biomarkers for intake of insoluble fibre, which limits our measures to provide a fully objective alternative indicator of adherence to recommendations and drinking powder. Even food sampling techniques with subsequent chemical analysis would need to be considered under the limitation of underestimated fibre contents [36, 37].

We could rule out a confounding effect of physical activity by using pedometers.

We experienced a one-year drop-out rate of 24% and additional 9% of subjects without reliable food records. These rates are within the range of those from other studies with lifestyle intervention or dietary supplements [13, 23, 38, 39]. Only few drop-outs due to incident T2DM occurred before the one-year visit in OptiFiT. Conducting our analysis for the full study period of two years would have meant to reduce the power to only 94 subjects, to accept a systematic bias by diabetes-related drop-outs, a lower compliance to lifestyle advice, supplementation and quality standards for our recurring food records. We therefore decide against an additional analysis of the full study period.

In summary, we demonstrate the effects of an intake of sufficiently high levels of insoluble fibre on the glycometabolic outcome in subjects with IGT. In average, intake of more than 14 g of insoluble fibre should lead to a reduction in capillary 2-h glucose levels, but an amount of 25 g should be aimed for in order to achieve the maximum benefit. Glycaemic improvements are accompanied by reduced insulin resistance, inflammation and potentially liver fat accumulation. Due to the supplementation design of the study, we can pinpoint the effect of healthy cereal products more convincingly towards the isolated component of insoluble fibre, irrespective of food matrix and additionally beneficial compounds. Both whole grain and fortified foods seem to be recommendable. Further targeted studies are required to assure replicability of our findings.

## Data Availability

Data sets are available by request to the corresponding author.

## Abbreviations

AA: Amino acid
ALAT: Alanine aminotransferase
ASAT: Aspartate aminotransferase
AUC: Area under the curve
BIA: Bioelectric impedance analysis
CRP: C-reactive protein
FLI: Fatty liver index
GGT: Gamma-glutamyl transferase
HIC: Hepatic insulin clearance
HOMA_IR_: Homeostasis model assessment insulin resistance index
IGT: Impaired glucose tolerance
ISI_ffa_: Insulin sensitivity index of blood free fatty acids
NAFLD: Non-alcoholic fatty liver disease
OptiFiT: Optimal Fibre trial for diabetes prevention
OGTT: Oral glucose tolerance test
T2DM: Type 2 diabetes mellitus
